# Mechanistic Insights into WO_3_ Sensing and Related Perspectives

**DOI:** 10.3390/s22062247

**Published:** 2022-03-14

**Authors:** Mauro Epifani

**Affiliations:** Istituto per la Microelettronica e i Microsistemi, IMM-CNR, Via Monteroni, 73100 Lecce, Italy; maurosalvatore.epifani@cnr.it

**Keywords:** WO_3_, chemoresistive sensors, sensing mechanisms, oxygen vacancies

## Abstract

Tungsten trioxide (WO_3_) is taking on an increasing level of importance as an active material for chemoresistive sensors. However, many different issues have to be considered when trying to understand the sensing properties of WO_3_ in order to rationally design sensing devices. In this review, several key points are critically summarized. After a quick review of the sensing results, showing the most timely trends, the complex system of crystallographic WO_3_ phase transitions is considered, with reference to the phases possibly involved in gas sensing. Appropriate attention is given to related investigations of first principles, since they have been shown to be a solid support for understanding the physical properties of crucially important systems. Then, the surface properties of WO_3_ are considered from both an experimental and first principles point of view, with reference to the paramount importance of oxygen vacancies. Finally, the few investigations of the sensing mechanisms of WO_3_ are discussed, showing a promising convergence between the proposed hypotheses and several experimental and theoretical studies presented in the previous sections.

## 1. Introduction

Tungsten trioxide (WO_3_) is currently attracting increasing attention as a material spanning several applications. Progress has been recently reviewed [1] on the electrochromic properties of WO_3_, one of the early notable features of this material [2]. The photochromic properties of WO_3_ have also been recently reviewed [3]. Throughout the years, other fields of interest have emerged, such as solar hydrogen generation [4,5], the photoelectrochemical [6,7] and photocatalytic [8,9,10] properties of WO_3_, water remediation [11,12,13] and energy storage [14,15]. Along with these recently emerging applications, the field of gas sensing was one of the earliest applications of WO_3_ together with electrochromic devices. While one the earliest reports on chemoresistive sensing with WO_3_ dates back to 1967 [16], interest in developing WO_3_-based sensors has since then steadily increased through early applications to H_2_S [17,18]. Currently, full reviews are devoted to the topic [19,20,21,22] (while of remarkable interest, WO_3_ sensors based on a gasochromic effect [23] will not be considered in the present work). On the one hand, the perusal of these reviews allows for the confirmation that a plethora of target analytes have been tested. On the other hand, a coherent picture of the factors governing sensing behavior has not yet emerged. In particular, the understanding of the sensing properties of WO_3_ is mainly based on the widely accepted concept of the modulation of the surface charge depletion layer [24] by the surface-adsorbed gaseous species. However, without a more detailed understanding of how and where the gaseous analytes react with the sensor surface, the depletion layer is more of a working hypothesis than an articulated model. Therefore, it seemed appropriate to devote a review work to the principles and issues related to WO_3_-based sensing to serve as an operative reference about the established facts, the known issues and possible further development pathways. This work is structured as follows. First, a succinct summary of some interesting results on WO_3_-based sensing will be presented. Then, the interconnection between structure, surface and surface defects in WO_3_, with particular reference to oxygen vacancies, will be discussed, as these are closely connected to the gas-sensing field. This discussion will be carried out by referring to both experimental findings and first principles studies. Finally, the few recent mechanistic studies on WO_3_ sensors will be discussed, showing that a fruitful convergence is appearing among fundamental studies of WO_3_ surfaces and the material’s gas-sensing properties. As a notice to the reader, noble metal loaded WO_3_ sensors are not the focus of the present review, despite the fact that several interesting results can be found in the literature [25]. Analogously, sensing [26,27] based on hexagonal [28,29,30] WO_3_ is a very timely topic but will not be covered in the present work. The reason is that such topics are, relatively speaking, in their infancy compared to pure WO_3_ in “conventional” phases.

## 2. Sensing Capabilities of WO_3_

WO_3_ is the second most studied gas-sensing material after SnO_2_ [31], and therefore it is not surprising that a large range of analytes have been tested for WO_3_-based detection. However, some general trends have been emerging, highlighting WO_3_ as a powerful sensor of NO_2_ and acetone in particular. Therefore, these two gases will be considered in more detail in the present section in order to evidence the physico-chemical properties of WO_3_ which are relevant for gas sensing. An additional paragraph will be devoted to the sensing of NH_3_ and amines, where WO_3_ seems in general to perform less well than in the case of the other two gases.

### 2.1. NO_2_

Most probably, the first study of WO_3_ as an NO_2_ sensor was published in 1991 by Yamazoe and coworkers [32]. The authors obtained a response of 97 at 300 °C to 80 ppm NO_2_. It was not possible to decrease the operating temperature due to exceedingly high electrical resistance attained below 300 °C. However, the obtained data allowed the authors to hypothesize that NO_2_ adsorption was responsible for the sensor response. We shall see in Section 4 that the most recent studies point to a reaction of NO_2_ with the surface oxygen vacancies. In a following paper, the authors evidenced the grain size effect on the device response [33]. Since then, materials research has focused on various features of the WO_3_ sensing layer. An important trend is the use of lamellar materials. Yamazoe and coworkers prepared WO_3_ lamellae [34] by acidification of Na_2_WO_4_ and found a response larger than 150 at 200 °C to 500 ppb NO_2_. Later on, Lee et al. [35] reported a response of about 960 to 5 ppm NO_2_ at 200 °C. The interest in such a morphology has not decreased through the years. For instance, Wang et al. [36] reported a response of almost 6 to 50 ppb NO_2_ at 140 °C. More recently, Wang et al. reported a response larger than 450 to 10 ppm NO_2_ at 100 °C [37]. The importance of having largely accessible surfaces was evidenced by Lee et al. [38], who used multi-shelled WO_3_ spheres to detect down to 50 ppb NO_2_ at 100 °C, with a response of about 100. It is clear from a more in-depth analysis of the literature, one which is reported in the following passages, that there is an interest in controlling the morphology of WO_3_ and obtaining high surface area structures.

You et al. [39] prepared monoclinic WO_3_ nanosheets assembled in microspheres by acid etching of CaWO_4_ hollow microspheres. They obtained a response of about 50 to 100 ppb NO_2_ at 75 °C. Akamatsu et al. [40] prepared monoclinic WO_3_ nanoplatelets assembled in microspheres by a surfactant-aided polyol process. A response of about 80 was obtained to 1 ppm NO_2_ at 200 °C. An et al. [41] reported an interesting monoclinic WO_3_ nanotube morphology prepared by TeO_2_ templating. A response of about 7 at 300 °C to 5 ppm NO_2_ was reported. Qi et al. [42] prepared monoclinic WO_3_ nanocolumns by a surfactant-free hydrothermal process. They reported the best operating temperature to be 110 °C, with a response of about 22 to 10 ppm NO_2_. Wang et al. [43] prepared monoclinic WO_3_ flower-like nanostructures by a surfactant-free solvothermal method. They obtained a response larger than 30 at 100 °C to 50 ppb NO_2_. Khan et al. [44] prepared monoclinic WO_3_ nanosheets by sonicating W powders in nitric acid, and could detect down to 40 ppb NO_2_ at 150 °C, with a response of 30. Harale et al. [45] prepared monoclinic WO_3_ nanobricks by the oxidation of metallic tungsten in H_2_O_2_. The resulting solution was used for the direct growth of the nanobricks onto glass substrates in autoclave conditions. The maximum response to 100 ppm NO_2_ was about 12, which, specifically, was obtained at 300 °C, while at lower temperatures a lower response was measured. Hua et al. [46] prepared monoclinic WO_3_ nanosheets by the acidification of Na_2_WO_4_. The thickness of the nanosheets was about 10 nm, much less than the width of the depletion layer in clean air conditions [33]. The authors supposed NO_2_ adsorption to be the basis of the sensing mechanism, and attributed the nonlinear behavior of the response at low NO_2_ concentrations (below 2 ppm) to competition with oxygen adsorption. A response of more than 32 was obtained at 300 °C to 4 ppm NO_2_. Beknalkar et al. [47] prepared 3D monoclinic WO_3_ nanoflowers and obtained a response larger than 50 to 100 ppm NO_2_ at 100 °C. Interestingly, Song et al. [48] observed that triclinic WO_3_ nanosheets prepared via the hydrothermal method displayed a larger response to NO_2_ than monoclinic and hexagonal WO_3_. They reported a response of 18.8 to 300 ppb of NO_2_ at 100 °C. Morais et al. [49] prepared monoclinic WO_3_ nanofibers by electrospinning and reported a response larger than four orders of magnitude to 50 ppm of NO_2_ at 150 °C.

Without detailing the results of an exceedingly long list of papers, we can notice some essential features with the help of Table 1. First of all, almost all the references report the formation of monoclinic WO_3_ after the heat-treatments necessary for thermally stabilizing the samples. Secondly, almost all the works report decreasing responses with increasing operating temperatures, supporting Yamazoe’s early model. Third, the influence of a porous accessible structure (lamellae, porous spheres, etc.) clearly emerges. The fourth point is that the authors of several papers increasingly indicate the importance of oxygen vacancies in determining the WO_3_ response to NO_2_ [50]. We will return to this last observation for a more in-depth discussion in the following passages. For now, we will proceed by perusing some relevant literature on acetone sensing.

### 2.2. Acetone

WO_3_ acetone sensors have been attracting special attention in the context of applications related to breath analysis [31,51,52], where humidity-independent acetone sensors are important for the monitoring of diabetes. However, WO_3_-based acetone sensing has a long history. In 2004 [53], Li and coworkers operated WO_3_ hollow spheres-based devices at 400 °C to detect 50 ppm of acetone with a response of about 3.5. In 2007, Khadayate et al. [54] used triclinic WO_3_ thick films for obtaining a response of 4.56 at 300 °C to 50 ppm acetone. In 2011, Chen et al. [55] used WO_3_ nanoplates to detect down to 2 ppm of acetone (a response of about 5) at 300 °C. Shi et al. [56] used monoclinic WO_3_ nanocrystals at 350 °C to get a response of 10 to acetone at a concentration of 10 ppm. Similarly to NO_2_, the interest in such morphologies has steadily increased, to the degree that, in 2012, Liu et al. [57] found 307 °C as the best operating temperature for WO_3_ nanoplates for detecting 300 ppm acetone, with a response of almost 50. In the same year, Chen and coworkers [58] used WO_3_ nanoplates with uneven surfaces to detect acetone at 200 °C (the response was about 40 to 500 ppm). Wang et al. [59] used nanoflowers composed of WO_3_ nanorods to detect 100 ppm of acetone at 300 °C (the response was 7). The interest in different morphologies has extended to nanotube-based devices, which could provide a response of 42.5 to 100 ppm acetone at 250 °C [60].

Chen et al. [61] used a hydrothermal process to prepare monoclinic urchin-like, rod-like and plate-like WO_3_. With urchin-like structures, they obtained a response of almost 15 to 25 ppm acetone at 300 °C. Zhang et al. [62] employed a hydrothermal process in varying acid conditions to prepare various crystallographic phases of WO_3_. However, after heat-treatment at 450 °C, monoclinic WO_3_ nanoplates were obtained. At an operating temperature of 300 °C, a response of about 8 to 100 ppm acetone was reported. A facet-dependent response was evidenced by Yin et al. [63], who reported a response of almost 50 to 100 ppm acetone at 340 °C, evidencing that the minimum nanoplate thickness was associated with the largest gas response among all the synthesized materials. Jia et al. [64] observed that triclinic WO_3_ nanosheets were more selective than monoclinic nanosheets, and detected 1 ppm of acetone at 230 °C with a response of 2.04. Xu et al. [65] demonstrated that monoclinic WO_3_ mesoporous nanofibers can be used to detect 50 ppm of acetone at 300 °C, with a response of 22, which is an improved result with respect to non-porous samples. Chang et al. [66] prepared WO_3_ nanosheets via the oxidation of WS_2_ nanostructures, and could detect 50 ppm acetone at 300 °C, with a response of about 15. As a final recent example, it is worth mentioning the work by Wang et al. [67], where sea-urchin-like monoclinic WO_3_ nanostructures were prepared using a hydrothermal method. A response of almost 30 to 100 ppm of acetone at 200 °C was reported.

From the analysis of these examples, whose features are summarized in Table 2, some trends emerge, as in the case of NO_2_. As expected, high operating temperatures are needed, typically ranging about 300 °C. There is a remarkably narrow range of responses for similar gas concentrations, which this indicates that acetone sensing is governed by the common features of the employed WO_3_ materials and does not depend on the synthesis process. Therefore, ubiquitous defects such as oxygen vacancies can be reasonably suspected to have a crucial effect on sensing properties.

### 2.3. Ammonia and Related Gases

While NO_2_ and acetone sensing applications span a large part of WO_3_-related publications, it is of interest to peruse the much less numerous applications devoted to ammonia and amines, since the detection of such gases may shed light on the adsorption properties of WO_3_.

In 1992, Yamazoe and coworkers [68] already investigated the ammonia-sensing properties of WO_3_. Au was needed for improving the response; the authors noted that the pure material had a response less than five to 50 ppm NH_3_ in the entire operation temperature range (200–600 °C). This limited response, and the need of additives such as Cr, was confirmed by Jimenéz et al. [69,70]. Later on, similar a response (5-6) to 50 ppm of ammonia was noted at about 250 °C [71]. Nguyen et al. [72] synthesized monoclinic WO_3_ nanowires and reported a maximum response of 9.7 to 1500 ppm NH_3_ at 250 °C. Therefore, a clear convergence can be observed among various papers, where the materials were prepared by different techniques (even orthorhombic WO_3_ nanofibers prepared by electrospinning were also studied, with similar results [73]), which report a limited response to ammonia. Such poor responses, and the consequent need of additives such as Cr [74], also helps to explain why WO_3_ has been seldom considered for ammonia sensing. Even more recent results with other material typologies, such as nanosheets assemblies [75] and nanoplates [76], are in line with the early papers. Despite the limited number of publications about the topic, the role of the surface sites occupied by additives [69] and the concentration of oxygen vacancies [74] has clearly emerged.

Ammonia related compounds, such as simple alkylamines, have been more efficiently detected than ammonia itself. What is likely the first report of trimethylamine (TMA) sensing below a concentration of 100 ppm concerned the use of WO_3_ hollow spheres: Lee et al. [77] could detect 5 ppm with a response of 56.9 at about 450 °C. Zhai et al. [78] used WO_3_ nanosheets self-assembled into microspheres to detect 50 ppm of triethylamine (TEA) at 220 °C, with a response of almost 16. Even Han et al. [79] reported low temperature TEA detection (150 °C) (a response of 35.3 to 10 ppm) using hierarchical WO_3_ spheres. Hu et al. [80] synthesized needle-shaped nanorods and reported optimum TEA sensing at 250 °C, with a response of about 50 to 50 ppm.

The summary in Table 3 shows that there is a rather relevant difference between the NO_2_/acetone couple and other analytes, such as ammonia and amines, with the latter providing much lower responses. This behavior suggests the presence of specific features in the surface of WO_3_ which make it more amenable to the adsorption of given gases. We are then prompted to have a closer look at WO_3_ surfaces and the related crystal structures.

## 3. WO_3_ Structures and Surfaces

### 3.1. Structural Complexity of WO_3_ and Relevant Hints for the Field of Gas-Sensing 

**Figure 1:**
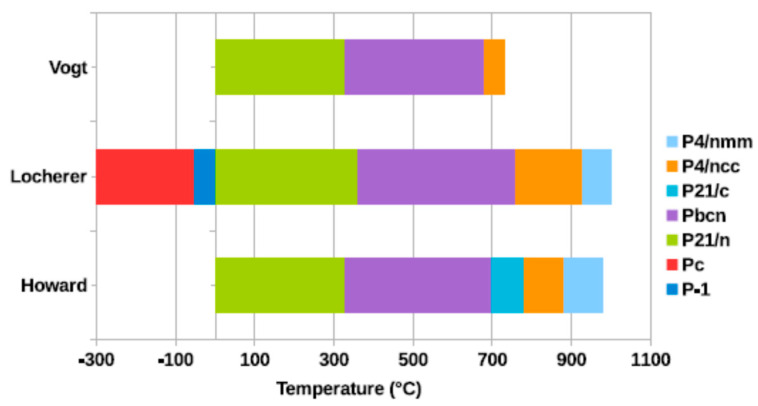
A visual summary of the crystallographic phase transitions in WO_3_, as obtained from the data published by the indicated authors. Reprinted with permission from [81]: H. Hamdi, E. K. H. Salje, P. Ghosez and E. Bousquet, Phys. Rev. B 94, 245124, 2016. Copyright (2016) The American Physical Society. License Number RNP/21/DEC/047874.

Saying “WO_3_” only is rather a generic reference. A complex system of phase equilibria is associated with the composition of simple trioxide [81,82,83]. Upon heating from a low temperature (up to −50 °C), phase transitions are reported, such as the transition from a monoclinic (ε-WO_3_) phase [84,85,86] to a triclinic (δ-WO_3_) one [83,87,88,89], and up to 17 °C, this is followed by another monoclinic phase (γ-WO_3_) [89,90,91]. An orthorhombic phase is found from 327 °C (β-WO_3_) [92,93] and is followed, from 740 °C, by a tetragonal phase (α-WO_3_) [92,94]. A visual summary of these phase transitions is given in Figure 1.

**Figure 2:**
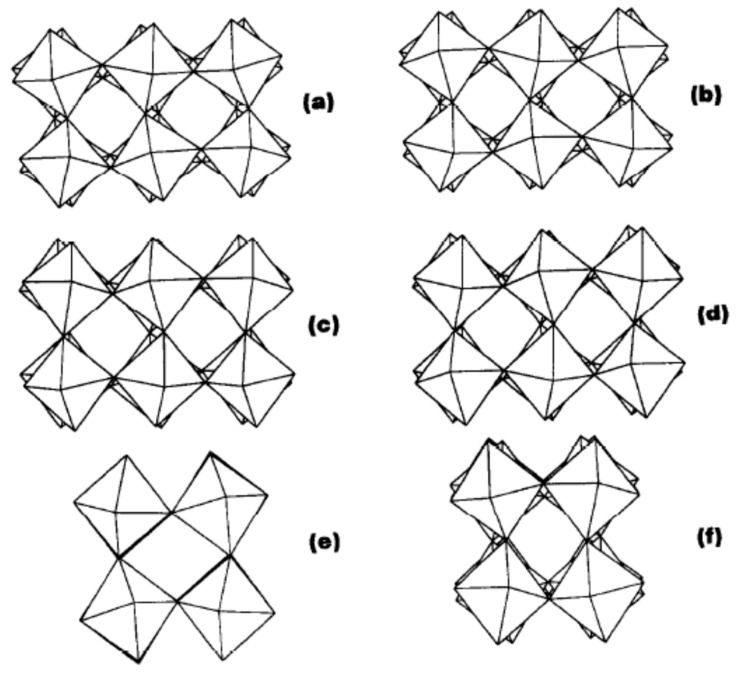
Tilt patterns of WO_3_: (**a**) monoclinic (001) projection, *x*-axis vertical; (**b**) triclinic (001) projection, *x*-axis vertical; (**c**) monoclinic (100) projection, *z*-axis vertical; (**d**) triclinic (100) projection, *z*-axis vertical; (**e**) monoclinic (010) projection, *z*-axis vertical; (**f**) triclinic (010) projection, *z*-axis vertical. Figure and caption reprinted from ref. [83]: J. Phys. Chem. Solids, Vol. 56, No. 10. P. M. Woodward, A. W. Sleight and T. Vogts, Structure Refinement Of Triclinic Tungsten Trioxide, pp. 1305–1315, copyright (1995) with permission from Elsevier. License number: 5223660238738.

A brief account of the historical development of structural investigations into WO_3_ has been provided by Howard et al. [95]. There are other phase transitions, but these are not discussed here for the sake of brevity, since they are observed at high temperatures (the interested reader can refer to the detailed description and references in ref. [96]). The reason for the complex structural behavior of WO_3_ can be understood if it is considered that WO_3_ possesses the ReO_3_ structure, where metal cations occupy the center of the oxygen octahedra sharing the corners. However, with respect to ReO_3_ (and to the perovskite structure from which both ReO_3_ and WO_3_ can be derived via removal of the A cation from the general ABO_3_ formula), WO_3_ features a whole set of possible distortions concerning both the octahedra relative positions (tilting) and the W cation position. In particular, as shown by Woodward et al. [83], the γ and δ phases are differentiated by the in-phase or out-of-phase tilting of the octahedra, respectively, as depicted in Figure 2. This close relationship between the two phases may partially explain their possible simultaneous presence in WO_3_ samples. It is interesting to note that ReO_3_ displays metallic conductivity, while intrinsic WO_3_ is an insulator (however, n-type conductivity is usually reported due to systematic oxygen non-stoichiometry [97]). In one of the earliest works devoted to the modeling of WO_3_, Stachiotti et al. [98] contributed to the explanation for differences between ReO_3_ and the hypothetic cubic structure of WO_3_, referring to the stability of the cubic structure upon lattice deformations. The translation of a metal cation along a lattice direction generates the hybridization of the O 2p and Re (d/t_2g_) orbitals (onset of π bonding between O and W), resulting in a level with a bonding contribution to the valence band and a level with an antibonding contribution to the conduction band. The latter would be occupied in ReO_3_, which is opposed to lattice distortion, so ReO_3_ is cubic. In WO_3_, such a level is empty and the structure distortion is stable. Since the removal of the A cation from the perovskite structure leaves an empty space at the center of the octahedron, foreign cations can be accommodated, giving rise to tungsten bronzes [99] (such as NaWO_3_). The latter also have an extra electron with respect to WO_3_, provided by the additional cation, and therefore they remain cubic, like ReO_3_, and are highly conductive. The cubic structure of WO_3_ was considered in ref. [98], analogously to earlier studies [100,101], for reasons connected to computational feasibility. However, cubic WO_3_ is not a stable phase due to the distortions described above, which are energetically preferred to cubic symmetry (such distortions are, in turn, a case of pseudo Jahn–Teller distortion [102]).

For this reason, increasing computational capabilities have prompted renewed interest in modelling the structure of WO_3_. De Wijs et al. [103] used a DFT (Density Functional Theory) methodology in the context of Generalized Gradient Approximation (GGA) for modelling several WO_3_ polymorphs. They confirmed that lattice distortions play a paramount role in determining the electronic structure of WO_3_ phases. In particular, the splitting of the W-O distances along different crystal axes is a fundamental phenomenological parameter that clearly evidences newly occurring electronic interactions. For instance, the simple modification from a cubic to a tetragonal structure involves alternate short and long W-O bonds along the *z* axis. The newly appearing overlapping of some of the W and O orbitals results in a bonding–antibonding interaction, with a strong influence on the valence and conductions bands. In the structures that are of major interest for sensors, i.e, the δ and γ phases, a similar phenomenon occurs, but this involves a splitting of the bond lengths along all the crystal axes. Wang et al. [104] used a hybrid functional to improve the estimation of the bandgap of WO_3_ polymorphs. Later on, Hamdi et al. [81] used a different hybrid functional in the context of DFT modelling to investigate the WO_3_ phase diagram and to understand the origin of the various phases on a microscopic scale. After ensuring that the calculated structural parameters agreed with previous studies, the next step was the modelling of the phonon spectra of the WO_3_ polymorphs. The authors demonstrated that the instability branches in the phonon spectra of cubic WO_3_ could be removed by the condensation of various modes of the cubic phase, and the newly arising phonon spectra were in very good agreement with the experimental polymorphs of WO_3_.

Even when only referring to these early considerations and studies, it should be made clear that properly considering the structures and defects of WO_3_ is a delicate and important task.

From this knowledge about WO_3_ structures, some operative considerations can be drawn that are of specific interest for the field of gas-sensing. First of all, monoclinic-orthorhombic transition occurs just above 300 °C. Therefore, any understanding of the sensing properties of WO_3_ cannot disregard the operating temperature of the device and the possibility that a different phase might be formed at the highest operating temperatures. There has recently been an experimental investigation of this issue, and this will be discussed in the final section. The second point concerns the very likely co-existence of δ and γ phases in the as-prepared samples.

**Figure 3:**
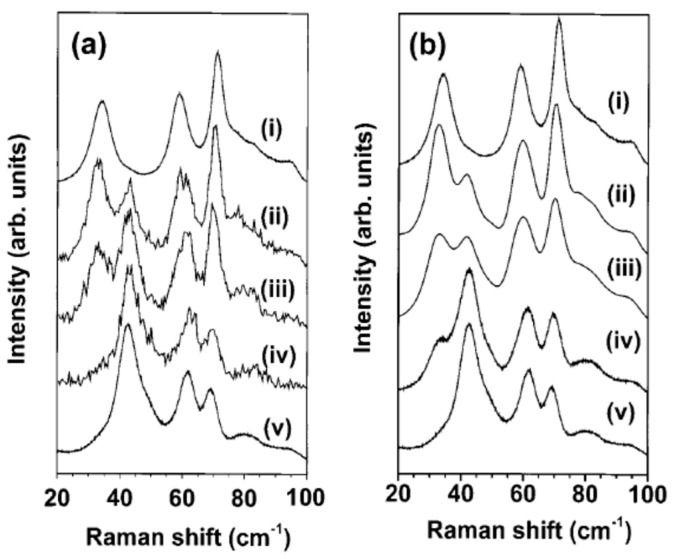
(**a**) Raman spectra of WO_3_ powder samples that were mechanically pressed for various time periods: (i) starting virgin powder (γ-phase); (ii) after 5 s compression; (iii) after 10 s compression; (iv) after 120 s compression; (v) reference treated powder sample, after 10 min moderate milling (δ-phase). Note the strong rearrangement of the low-frequency spectra after the early steps of pressure application. (**b**) Low-frequency Raman spectra collected at RT on WO_3_ virgin powder rapidly cooled by immersion in liquid nitrogen and kept there for different time periods: (i) starting virgin powder; (ii) after 5 s immersion in LN_2_; (iii) 1 min immersion; (iv) 10 min immersion; (v) 2 h immersion. The varying intensity ratio between the peaks at 34 and 43 cm^−1^ is clearly visible. Reprinted from ref. [106]: J. Solid State Chem., Vol. 143, E. Cazzanelli, C. Vinegoni, G. Mariotto, A. Kuzmin, and J. Purans, “Low-Temperature Polymorphism in Tungsten Trioxide Powders and Its Dependence on Mechanical Treatments”, pp. 24–32, Copyright (1999) with permission from Elsevier. License number: 5225870445333.

As described in detail by Woodward et al. [83], the boundary between the two phases is not sharp; while the transition from the triclinic δ phase to the monoclinic γ phase is generally accepted to occur at 17 °C, the triclinic form seems to be the most stable one at room temperature, where it is indefinitely stable, while the monoclinic phase is converted to the triclinic upon grinding at 25 °C. In practice, it cannot be excluded that a mixture of the two phases is present in the prepared samples after cooling to room temperature [105,106,107]. In principle, the two phases can be distinguished by X-ray diffraction. However, as observed by Jimenez et al. [108], the envelope of reflections to be used for discriminating the phases, placed at a 2Θ value of about 34°, may fail to provide a clear response due to instances of peak broadening in the case of nanocrystalline samples. The authors employed a method proposed by Cazzanelli et al. [106] (this reference also quotes an earlier paper where the different Raman spectra between the two phases was reported) based on Raman spectroscopy, since the monoclinic phase displayed a band at 34 cm^−1^, while, for the triclinic one, the band was at 43 cm^−1^. In Figure 3, the clearly-distinguished Raman spectra for the two phases are shown, as originally reported in ref. [106].

These considerations should have clarified that the determination of the structure of WO_3_ is a delicate issue. Neglecting the complex phase transitions system may result in an inexact starting point for understanding the material’s sensing properties. This issue is even more relevant, currently, due to the above-mentioned interest in faceted materials whose final morphology clearly depends on the crystallographic phase. In turn, the exposed facets also impose which are the most relevant surface defects (e.g., which kind of oxygen vacancies) to be considered in the gas sensing phenomenon. Therefore, the next step of the present discussion will be devoted to the accumulated knowledge on the surface and defects of WO_3_ depending on the crystallographic phase.

### 3.2. Oxygen Vacancies in WO_3_

#### 3.2.1. Experimental Findings: Surface Oxygen Vacancies

##### Electrical Properties

The importance of oxygen vacancies for the electrical properties of WO_3_ has been known for long time. In as early as 1970, Berak and Sienko [97] reported an increase in the carrier concentration upon oxygen removal from the triclinic/monoclinic phases in WO_3_ single crystals. The dependence of the electrical properties of WO_3_ on oxygen stoichiometry has been a steadily recurring observation throughout the years. For instance, Kaneko et al. [109] reported that the resistivity of sputtered WO_3_ thin films remarkably depended on the oxygen concentration in the sputtering atmosphere. A critical oxygen concentration of 4–5% was reported, beyond which transparent films were obtained. The resistivity of the films increased by several orders of magnitude with respect to a sputtering atmosphere with a slightly lower oxygen concentration. Gillet et al. [110] attributed to oxygen vacancies the conductivity of sputtered WO_3_ thin films, on the basis of resistivity measurements carried out in different oxygen concentrations. Interestingly, they argued that the observed decrease in conductance activation energy with a decrease in oxygen partial pressure was due to the overlap of increasingly dense defect states, finally resulting in an energy band in the WO_3_ gap. Its width, which increased with increasing defect concentrations, would finally result in the disappearance of the gap. Li et al. [111] prepared γ-WO_3_ films via spray processing and investigated the effect of the annealing atmosphere on the electrical conductance. They, too, observed a conductance increase upon annealing in N_2_ and a decrease upon annealing in 95% N_2_-5% O_2_, which were attributed to generation and the healing of oxygen vacancies, respectively. It was only after annealing at temperatures higher than 600 °C that additional conduction mechanisms were supposed.

##### Spectroscopic Studies

Early evidence was obtained by X-ray photoelectron spectroscopy (XPS) of the existence of several W oxidation states in WO_3_ in the context of the electrochromism of amorphous WO_3_ thin films. It is worth providing a brief historical sketch of the development of the spectral interpretation. Hollinger et al. [112] observed an additional signal in the valence band spectra of UV irradiated, colored amorphous WO_3_ thin films. They argued that the band should originate from W5d states, i.e., conduction band states that are empty in intrinsic WO_3_. The population of such states was parallel to the appearance of a W(V) signal in the W4f core levels spectra. De Angelis and Schiavello [113] reported a similar signal in the valence band of several substoichiometric tungsten oxides, again evidencing the filling of the W5d states. In 1978, Colton et al. [114] explicitly connected oxygen removal from WO_3_ thin films to the appearance of a small band in the valence spectra. Any additional electron resulting from oxygen removal would occupy the normally empty (in a purely ionic description of the WO_3_ crystal) W5d states, which would mean the reduction of W(VI) to W(V). The following year, Salje et al. [115] had to use several components to fit the XPS spectra of WO_3_ single crystals reduced by ion bombardment. In 1981, Bringans et al. [116] investigated the (001) surfaces of monoclinic WO_3_ by ultraviolet photoemission spectroscopy (UPS). They observed that, upon bombardment of the surfaces with electrons, argon ions and oxygen ions, a density of states appeared in the spectra in the energy gap. By referring to previous studies, where the preferential removal of oxygen had been observed for several oxide surfaces, the authors concluded that the newly appearing density of states was due to generated oxygen vacancies. In a following paper, Höchst and Bringans [117] again attributed to oxygen vacancies the appearance of a density of states in the energy gap of WO_3_ thin films grown by evaporation. When the films were annealed at increasing temperatures, the peak increased and the films finally became metallic. Exposure to atomic oxygen resulted in the disappearance of the peak and, following an interpretation of the peak, it was determined that this was due to the presence of oxygen vacancies. Since then, oxygen vacancies and their spectroscopic signature through several W states have progressively become accepted. In a particularly clear and important example, Dixon et al. [118] carried out a series of investigations on WO_3_ surfaces using several techniques. In one of these papers, this group employed resonant photoemission to investigate the (001) surface of cleaved crystals of monoclinic WO_3_, with the photon energy tuned to resonance with the W5d states.

**Figure 4:**
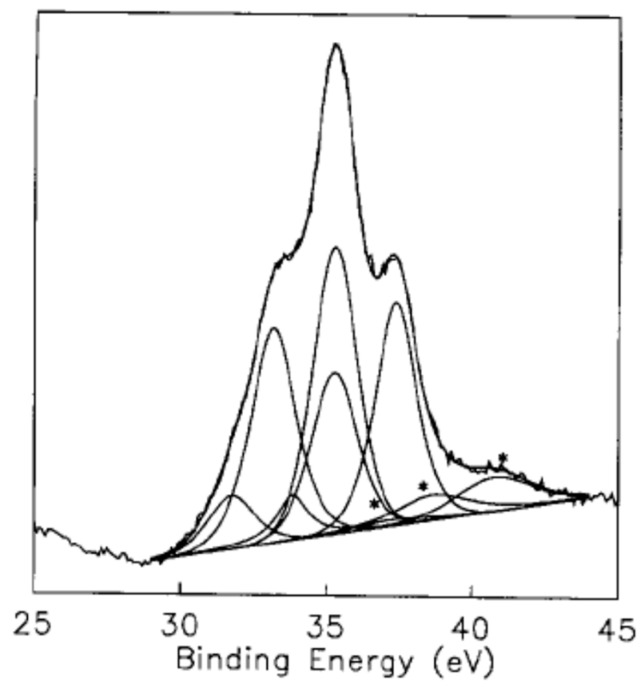
Fitting of the W4f energy region. The stars indicated the 5p_3/2_ which had to be added to the 4f_7/2_ + 4f_5/2_ doublets for a correct fit of the experimental curve. Reprinted from Figure 6a in Surf. Sci., Vol. 399: R.A. Dixon, J.J. Williams, D. Morris, J. Rebane, F.H. Jones, R.G. Egdell, S.W. Downes, “Electronic states at oxygen deficient WO_3_(001) surfaces: a study by resonant photoemission”, pp. 199–211, Copyright (1998), with permission from Elsevier. License number: 5235331217794.

After mild ion bombardment, a W5d structure clearly emerged, attributed to oxygen removal from the surface and the consequent filling of the otherwise empty W5d band, in agreement with the early studies reported above. The analysis of the W5d region (valence band spectra) after prolonged bombardment showed complex spectra featuring several components, attributed to 5d^0^, 5d^1^ and 5d^2^ states, the last two being associated with the effects of ion bombardment. Prolonged ion bombardment resulted in the enhancement of the described spectral features. In particular, the W5d band resulted in a much higher density of states at the Fermi level, with the pronounced metallic nature of the surface. Analogously, the XPS W4f core level presented a complex structure after ion bombardment, requiring three doublets for a correct fit. Such doublets were associated with the appearance of the 5d^0^, 5d^1^ and 5d^2^ components in the valence band spectra, and therefore were associated with the reduction of the surface undergoing the bombardment. The authors could therefore associate different surface structures to the bombardment intensity. This achievement represented progress in terms of the knowledge of the surface chemistry of WO_3_ with respect to early works, where complex XPS spectra had already been interpreted in terms of oxygen vacancies. The importance of the W5p_3/2_ component in the fitting of the W4f region was also evidenced. An example of such fittings is reported in Figure 4, where the whole set of oxidations states, from W(IV) to W(VI), and the W5p_3/2_ component, were used. As a final example, the photoemission study on WO_3_ polycrystalline thin films by Bussolotti et al. can be quoted [119]. Even in this case, the presence of oxygen vacancies, whose concentration could be modulated by annealing in ultra-high vacuum, was associated with the complex line shape of the W4f photoemission signal.

**Figure 5:**
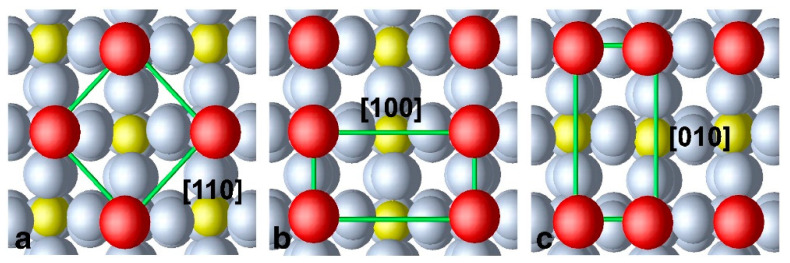
Top view of O-terminated surface for the reconstructions: (**a**) ($\sqrt{2}\times \sqrt{2}$ R45°, (**b**) (2 × 1) and (**c**) (1 × 2). Red spheres are surface oxygen atoms, while yellow and gray spheres are tungsten and other oxygen atoms, respectively. Reprinted from Surf. Sci., Vol. 606: C. Lambert-Mauriat, V. Oison, L. Saadi, K. Aguir, “Ab initio study of oxygen point defects on tungsten trioxide surface”, pp. 40–45, Copyright (2012) with Permission from Elsevier. License number: 5227571391232.

Another interesting technique which involves directly visualizing the structure of reduced WO_3_ surfaces is scanning tunneling microscopy. Jones et al. [120] studied the structure of the (001) surface of cleaved monoclinic WO_3_ crystals before and after ion bombardment and annealing in UHV conditions. They argued that, in the stoichiometric (001) surface, half of the oxygens are missing in order to avoid the situation of a surface with divergent energy (Tasker’s rule). This requirement results in the experimental observation of surface $\sqrt{2}\times \sqrt{2}$ R45° reconstruction. Such surfaces easily lose oxygen ions, and the defects organize in linear structures, with the W ions adjacent to which assuming the W(V) state. Figure 5 shows models of the possible reconstructions of the (001) monoclinic surface.

#### 3.2.2. First Principles Modeling of Oxygen Vacancies in WO_3_

The ubiquitous appearance of oxygen vacancies, and their impact on the physical properties of WO_3_, have prompted widespread interest in the understanding of the origin and electronic properties of such defects. While the γ and δ phases are the main interest in terms of gas sensing, several thorough theoretical investigations of various polymorphs exist. Once again, the simple cubic structure was considered in early studies [121,122]. Later on, Chatten et al. [123] employed the Perdew–Becke–Ernzerhof functional in the DFT framework for studying several WO_3_ polymorphs, both intrinsic and oxygen-deficient. The structural relaxation after introducing an oxygen vacancy resulted in increased WW distance across the vacancy and in a modification of the WO bond length splitting along all the directions. Such modification resulted in new bonding–antibonding interactions with different effects depending on the considered direction. They also established that for all the studied polymorphs, the lowest bands of the W5d levels (which in WO_3_ roughly gives rise to the conduction band, while the valence band is mainly contributed to by the O2p levels) crossed the Fermi level, so the systems had a metallic nature. This is one of the still most intensely debated topics in investigations of the electronic structure of WO_3_ and is of outmost importance since it is closely related to the understanding of electrochromism in WO_3_ [124]. Later on [125], Lambert-Mauriat and Oison [125] used both LDA and GGA functionals to study the oxygen vacancies of γ-WO_3_. They confirmed the metallic nature of the oxygen-deficient phase due to the filling of the bottom of the conduction band and, hence, to the partial filling of the W5d states contributing to it. Interestingly, they considered different vacancies depending on the specific oxygen removed from the considered cell. Indeed, in the monoclinic structure there are three non-equivalent oxygens, depending on the crystallographic direction. In 2011, Wang et al. [126] argued that the metallic nature of oxygen-deficient WO_3_ could be due to the use of standard functionals in DFT, resulting in the underestimation of the band gap and therefore resulting in the aforementioned position of the Fermi level in the conduction band. For this reason, they used hybrid functionals in the DFT frame, together with the distinction of the oxygen vacancies depending on the crystallographic direction. They established that a semiconductor-to-metal transition occurs with an increase in the concentration of the oxygen vacancies. For WO_2.94_ (diluted case), the defects gave rise to a band clearly localized into the band gap, with a large separation from the bottom of the conduction band in the case of vacancies formed along all the axes. For WO_2.88_, the levels of the vacancies formed along the *x* and *y* axes crossed the Fermi level. For the vacancies along the *z* axis, the system remained semiconductive. Since the formation energy of the vacancies was independent from the direction in the lattice, it could be argued that a population formed by the three kinds of vacancies could have been expected. This approach was refined later on [127] by using a larger supercell and a different functional. In this more diluted vacancy configuration, the authors found that the vacancy formed along the *x* axis is strongly localized in the vacancy void. The vacancies along the other axes feature a triplet ground state with a shallower nature with respect to the vacancy on the *x* axis. The excess charge was distributed among the W5d states along the WOW chain. Above all, the vacancy on the *z* axis had a clear conductive nature, which was interpreted as the basis of the always-invoked relationship between WO_3_ electrical conductivity and oxygen vacancies. Mehmood et al. [128] and Migas et al. [129] confirmed merging of the vacancy levels with the conduction band in the case of cubic and γ-monoclinic WO_3_. It can be seen how complex investigations into the physical properties of WO_3_ can be and how they critically depending on the chosen model.

**Figure 6:**
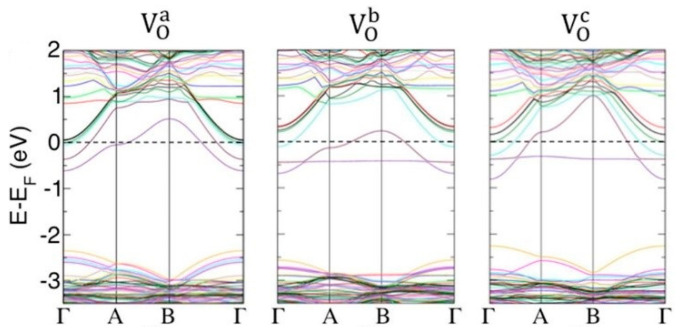
Electronic structure of defective WO_3_ surfaces with oxygen vacancies formed along the indicated axes. The Fermi level is represented by a horizontal dashed line at E-E_F_ = 0. Reprinted with permission from Figure 3a in ref. [132]: E. Albanese, C. Di Valentin, Gianfranco Pacchioni, “H_2_O Adsorption on WO_3_ and WO_3−x_ (001) Surfaces”, ACS Appl. Mater. Interfaces 2017, 9, (27), 23212–23221. Copyright (2017) American Chemical Society.

Until now, bulk oxygen vacancies have been considered. Such investigations are instructive since they concerns the complex interplay between structural and electronic properties in WO_3_. However, it is more immediate in the context of the field of chemosensors field to consider surface vacancies. In this case, an increasing number of studies have been appearing in the literature. This interest is prompted by the use of WO_3_ in water splitting and other energy related applications. Lambert-Mauriat et al. [130] investigated the oxygen vacancy formation on the (001) surface of γ-WO_3_ using DFT. Such a surface was calculated to be the most energetically favorable, and this was later confirmed theoretically [131] and was in agreement with previous experimental evidence [120]. They found that the vacancies have an n-doping effect. This result was attributed to the band gap underestimation by the employed DFT functionals. Another important conclusion was that the adsorption of oxygen was more energetically demanding than the formation of the vacancy, which explains the stability of oxygen-deficient WO_3_ in various oxygen atmospheres. The same surface was considered by Albanese et al. [132], who also took into account the non-equivalence of the oxygen species (depending on the crystallographic axes) to be removed for forming a vacancy. They found that the three kinds of vacancies result in defect levels merging with the conduction band (see Figure 6). This is a very important conclusion, corroborating the early experimental attribution of the electrical properties of WO_3_ to surface oxygen vacancies. Moreover, the formation of the oxygen vacancy at the *c* axis was more favorable than the bulk value (with the formation energy lowered by 1.5 eV), justifying the expected non-stoichiometry of annealed surfaces.

Summarizing:

1. Oxygen vacancies (both in bulk and on the surface) in WO_3_ are ubiquitous and favorably formed during the heat-treatment. Re-healing is not a favorable process, with WO_3_ tending to remain oxygen-defective;
2. There is theoretical evidence that oxygen vacancies are anisotropic, with the formation energy depending on the particular crystallographic axis. However, a distribution of all the kinds of vacancies can be expected. The vacancies confer electrical conductivity to WO_3_. The most energetically favorable surface of monoclinic WO_3_ is the (001).

#### 3.2.3. Surface Oxygen Vacancies in WO_3_ and Adsorption Properties

##### Experimental Studies

The catalytic properties of reduced tungsten oxides were established several years ago [133]. An important feature of WO_3_ surfaces is the presence of strong Lewis sites, which play an essential role in the adsorption of a gaseous species in catalysis and, therefore, are of paramount importance for sensors as well [134].

Ramis et al. [135] prepared a “pseudocubic phase” of WO_3_ by processing ammonium paratungstate. The investigated samples were initially a mixture of the hexagonal and a “pseudocubic phase”. The latter was converted after heat-treatment at 748 K to what was described as a “poorly crystallized pseudocubic phase”. The phase determination was uncertain between the monoclinic and orthorhombic WO_3_. The investigation of the surface sites was carried out by the adsorption of molecular probes followed by FTIR analysis after evacuation of the samples in the FTIR cell at a maximum temperature of 373 K. Evidence was provided for very strong Lewis sites, which were identified by the authors as surface W^6+^ ions, coordinatively unsaturated. Later on, Kanan et al. [136], using similar techniques, investigated the surface of monoclinic WO_3_. They confirmed the Lewis acidity of the surface and, with respect to ref. [135], they could evacuate the sample at 400 °C. However, a decrease of the Lewis sites by 50% was already observed after evacuation at 150 °C, which was attributed to surface reduction. Instead, adsorbed water did not seem to play any role in the modification of the Lewis sites. The role of the acidic W surface sites was confirmed by the following desorption studies.

Using calibrated thermal desorption spectroscopy, Ma et al. [137] studied the desorption of methanol from both oxidized and reduced γ-WO_3_ (001) thin film surfaces. They found that, on surfaces oxidized by heat-treatment at a high temperature, methanol was adsorbed onto strongly acidic sites, probably coordinatively unsaturated W(V) sites. No dissociation of the molecule occurred. In contrast, methanol dissociation occurred over the reduced surfaces. The authors attributed this behavior to the increased basicity of the bridging oxygens onto the reduced surface. Such oxygen species would be involved in the dissociation step together with the W(V) sites that are the adsorption site for methanol. In a later paper, Ma and Frederick [138] investigated the desorption of ethanol and 2-propanol from the same surfaces. They found that desorption from the reduced surfaces occurred at higher temperatures than for oxidized surfaces at any coverage value. At lower temperatures, the desorption products were alcohol molecules, while a second desorption channel generated alkenes and water. Water desorption was also investigated and, again, the desorption temperature was higher for reduced surfaces than for the oxidized ones. Water dissociation was not observed.

##### First Principles Modeling of Adsorption onto WO_3_ Surfaces

In 2011, Ling et al. [139] used the Perdew–Burke–Ernzerhof (PBE) functional to investigate the adsorption of hydrogen (atomic and molecular) and methanol onto various surfaces, among which defect-free (001) ε-monoclinic WO_3_ was included. They considered as adsorption sites two oxygen sites (terminal O_1C_ and bridging O_2C_) and the coordinatively unsaturated W_5c_ site. The authors reported more favorable atomic hydrogen adsorption onto the oxygen sites, with comparable adsorption energies. In the case of molecular hydrogen, dissociation onto O_1C_ or onto O_1C_ + O_2C_ was found to be the only energetically favorable process. The most favorable outcome was hydrogen dissociation onto the O_1C_ + O_1C_ sites. The authors also considered desorption of a water molecule from the surface (water results from the recombination of the hydroxyl groups formed from hydrogen dissociation) which resulted in surface reduction. In the case of methanol, no dissociation was predicted, and this was in agreement with the aforementioned experimental studies. Unfavorable methanol dissociation onto the γ-monoclinic (001) surface was confirmed later on [140] by the same group. In 2012, Wang et al. [141] re-examined the problem of hydrogen adsorption by considering the γ-monoclinic (001) surface and implementing the DFT scenario with the hybrid B3LYP functional. The reason for this was the need to avoid bandgap underestimation using the standard DFT methods. In the case of atomic hydrogen, the authors considered several surface oxygen configurations. For 1-coordinated, top O_1t_ oxygen, they found that the proton releases an electron to the nearby W atom, giving rise to a state 0.44 eV below the bottom of the conduction band. In the case of the newly formed H-O bonds onto the in-plane oxygen atoms, this resulted in more delocalized states which were, to different extents, merged with the conduction band. The adsorption energies were similar for all the considered configurations, suggesting the favored migration of the proton over the surface. In the case of molecular hydrogen, the various considered configurations gave rise to endothermic dissociation processes, in contrast with ref. [139]. The authors attributed the discrepancy to several factors, including the different functional employed in DFT implementation and the different WO_3_ crystallographic phase. Later on [132], the same group investigated the adsorption of water onto the stoichiometric and oxygen deficient (001) surface of γ-monoclinic WO_3_. They found that, on the perfect surface, the adsorption of molecular water is favored over dissociation, and that the latter is favorable only for high surface coverage. In such a case, the surface is completely covered by a network of hydrogen bonded W-OH groups. In the case of the oxygen defective surface, the authors distinguished among various vacancy topologies, as discussed at the end of Section 3.2.2. They did not find relevant differences with respect to the stoichiometric surface, with adsorption still favored with respect to dissociation. Interestingly, the authors reexamined the problem of hydrogen dissociation onto defective WO_3_. In fact, the final water configuration after adsorption is equivalent to adsorbing hydrogen onto the W_5c_ site of a stoichiometric surface (the water oxygen refilling the oxygen vacancy). With respect to their previous work [141], they found exothermic hydrogen dissociation onto terminal, 1-coordinated O_1c_ ions. Heterolytic dissociation was less favorable. The discrepancy was explained on the basis of the small cell previously used. The most important conclusion was that, if water desorption could be modeled as hydrogen dissociation, and the latter, as evidenced by the authors, introduces states merged with the conduction band, the overall effect of water desorption is surface reduction. On the other hand, Zhang et al. [142] confirmed that water adsorption is more favorable than dissociation onto the stoichiometric, monoclinic WO_3_ (001) surface, with the energy difference between the two processes decreasing with increased water coverage. However, diverging from previous studies, they found that dissociation is an exothermic process onto defective surfaces. Finally, Hurtado-Aular et al. [143] recently confirmed that water adsorption is more favorable than dissociation over stoichiometric (001) and (100) γ-monoclinic surfaces. Dissociation was more and more favored with increasing surface coverage by water molecules. The perusal of these studies shows the difficulties involved in modeling the surface chemistry of oxides such as WO_3_.

Summarizing:

1. Experimentally, the surface of monoclinic WO_3_ contains strongly acidic W sites; the reduction of the surface strongly favors adsorption of methanol and water. Methanol dissociation takes place on reduced surfaces, which is not the case with water.
2. Theoretically speaking, water adsorption is generally calculated to be more favorable than dissociation. However, water desorption resulting from hydrogen dissociation results in surface reduction. We can take this result as an indication that, reciprocally, water dissociation on reduced WO_3_ surfaces may result in the healing of oxygen vacancies. While apparently unfavorable in the DFT scenario, water dissociation cannot be neglected from experimental studies where an electrical bias is also applied, such as in the case of chemoresistive sensors.

## 4. Mechanistic Studies of WO_3_ Sensor Properties

The study of sensing pathways with WO_3_ based devices is much less developed than for SnO_2_, despite an increasing number of works dealing with WO_3_ sensors, as briefly seen in Section 2. Therefore, the aim of this section is to review the available results and, finally, to try to connect state of the art studies with the previous sections, highlighting possible further developments in terms of pursuable research lines and potentially applicable methodologies.

**Figure 7:**
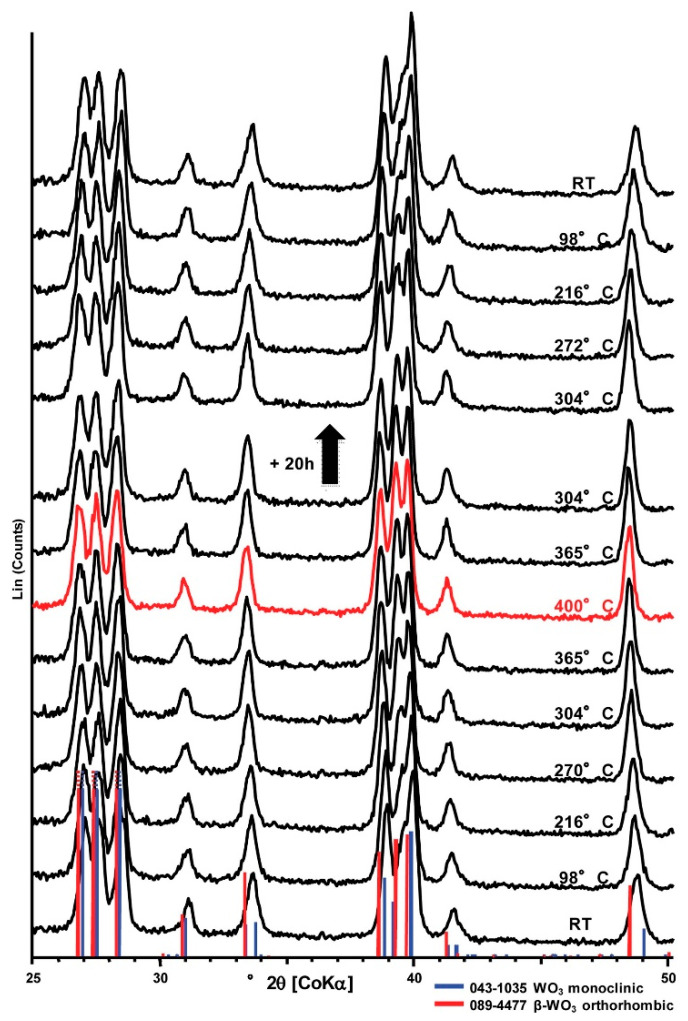
XRD patterns recorded on a WO_3_ sample during in situ heating up to 400 °C, followed by holding for 20 h at 304 °C and cooling to room temperature. The phase transition from monoclinic to orthorhombic at about 300 °C is clearly visible. Reprinted from Sens. Actuators, B: Chem., Vol. 237, A. Staerz, C. Berthold, T. Russ, S. Wicker, U. Weimar, N. Barsan, “The oxidizing effect of humidity on WO_3_ based sensors”, pp. 54–58, Copyright (2016) with permission from Elsevier. License number: 5247560728197.

The first mechanistic study was published in 2010 by Hubner et al. [144]. The authors performed CO sensing measurements both in presence and absence of oxygen, and simultaneously recorded CO_2_ production in the cell exhaust. Importantly, the authors also performed measurements in the absence of CO for the purpose of excluding any catalytic effect from the Pt electrodes. This issue is as important as oversight in sensor studies and should be more systematically assessed. The response to CO in the absence of oxygen (O_2_ concentration < 3 ppm) ranged over one order of magnitude for CO concentrations from 10 to 100 ppm. The authors concluded that surface reduction by CO occurrred, according to the following equation:

(1) $$CO^{gas}+O_{O}\to CO_{2}^{gas}+V_{O}^{•}+e^{-}$$

where *CO^gas^* is the target gas, *O_O_* is a regular oxygen lattice site, *CO_2_^gas^* is the gaseous by-product, *V_O_^●^* is a singly ionized oxygen vacancy and e^−^ is the electron released to the conduction band, finally increasing the conductance. Slow recovery was observed, attributed to surface re-oxidation with the residual oxygen in the cell. This behavior was markedly different from that of SnO_2_, where direct reduction of the surface by CO was not observed. From this early study, we can already conclude that the concentration of oxygen vacancies is of paramount importance in determining the WO_3_ sensor response. With an increase in the vacancy concentration, the surface will oppose more and more to further reduction, therefore hampering the sensor response. From the previous discussion, we know that the surface of as-prepared WO_3_ typically contains oxygen vacancies and that this is an energetically favorable surface configuration. Therefore, planning to use a perfectly stoichiometric WO_3_ surface would result in s high response due to the availability of the maximum possible concentration of removable oxygens. However, full recovery would not be a favorable process, thus hampering the repeatability of the performance. In 2016 [145], the same group investigated another important difference with respect to SnO_2_ sensing. While exposure to water vapor has been known for long time to decrease the electrical resistance of SnO_2_ [146], the authors had already observed an opposite effect for WO_3_ [147]. First of all, an in situ XRD study was conducted to verify the crystal phase stability of the tested γ-monoclinic WO_3_ devices at the operating temperatures. A reversible monoclinic → orthorhombic was observed at about 300 °C during the heating stage, which was the operating temperature of the sensors. Upon cooling, the reverse transition occurred at about 272 °C. This essential study, an example of which is reported in Figure 7, is probably one of the very few examples for WO_3_. The importance of a correct phase determination for understanding the sensing properties of WO_3_ has already been outlined in Section 3.1. The second result of the study was the confirmation of a decrease in electrical resistance with increasing levels of humidity at 300 °C. It was shown that, in agreement with their previous study, humidity did not interfere with the CO operation (the sensor signal increased) while a decrease in the NO_2_ response was observed with increasing humidity. Finally, by operando DRIFT (Diffuse Reflectance Infrared Fourier Transform) spectroscopy the authors investigated the surface reactions of CO and NO_2_ with the devices during the sensing operation. The conclusion was that CO resulted in surface reduction by extracting oxygen from WO_3_, while NO_2_ healed the surface oxygen vacancies, thus decreasing the signal. Very importantly, the authors concluded that the effect of humidity was due to vacancy healing by the oxygen contained in the water molecule, in perfect agreement with the conclusions of the first principles studies discussed in the previous section (there, water desorption resulted in surface reduction). However, the experimentally determined WO_3_ phase (orthorhombic) in ref. [145] was different from that in ref. [132]. This additional issue was considered in a following work [148] by the same group, where experimental DRIFT studies were coupled with first principles DFT modeling onto the orthorhombic WO_3_ surface. In this complex experimental case, where the concurring adsorption of oxygen species was also demonstrated, the authors also confirmed that the increase in the resistance of the sensors at 300 °C was due to water adsorption on the surface, resulting in the healing of oxygen vacancies and hydrogen generation. Hydrogen production was also measured experimentally. Finally [149], such sensing properties of WO_3_ were demonstrated to be independent of the synthesis process and, overall, closely connected with the presence of oxygen vacancies. This last conclusion gave rise to a negative outlook regarding the achievability of selective sensors.

The experimental findings on the CO reaction mechanism were confirmed early on by DFT modeling [150], where the authors confirmed direct surface reduction by CO and the formation of oxygen vacancies. Instead, in the same study, ozone sensing was predicted on the basis of oxygen vacancy healing upon the dissociation of the ozone molecules. In the case of NO_2_, Saadi et al. [151] showed that, in agreement with the previously mentioned experimental findings, the likely pathway consists in oxygen vacancy healing by NO_2_, implying an increase in electrical resistance.

## 5. Conclusions

The present review work has attempted to critically analyze several aspects of the physics and chemistry of WO_3_ related to the sensing properties of this material. Considerable obstacles remain to be overcome, however. Therefore, in the conclusions, the established facts will first be mentioned, and possible further directions will then be briefly indicated.

The following noteworthy established points emerged:

1. The complex crystallographic phase diagram of WO_3_ must be taken into account when determining the phase involved in the sensing activity. At room temperature, the as-prepared samples may be constituted by a mixture of γ-monoclinic and δ-triclinic phases, whose speciation can be facilitated by combining XRD and Raman spectroscopy. The investigation of the sensing behavior cannot disregard that at typical operating temperatures, such as 300 °C, the active phase may be the orthogonal one. Therefore, determining the phase composition of the as-prepared samples can be insufficient for studying sensing properties.
2. The oxygen vacancies dominate the physical properties of WO_3_. From first principles modeling, it appears that both bulk and surface vacancies share some features: the formation energy and the electronic effect of the vacancies depend on the crystallographic direction along which they are formed. However, an increasingly accepted view is that at least some of the vacancy levels merge with the Fermi level, explaining the electrical conductivity of oxygen-deficient WO_3_, in agreement with experimental findings. From an experimental point of view, XPS analysis is extremely useful in distinguishing the various W oxidation states connected to the presence of oxygen vacancies. However, such a technique must be used carefully. For instance, the fitting of the W4f region cannot exclude a W5p_3/2_ component. Analysis of the valence band spectra may provide useful information about the presence of oxygen vacancies.
3. Experimental studies into the sensing mechanisms of WO_3_ have radically modified the common view of chemoresistive sensing. In the case of CO sensing, the direct generation of oxygen vacancies onto the WO_3_ surface has been solidly supported. In the case of NO_2_, the healing of surface vacancies with NO by-product has been backed up by considerable evidence. These conclusions deeply question the usual hypothesis regarding the charge depletion layer by oxygen adsorption and its subsequent modulation by the gas analyte. This is a general indication to carefully check for the applicability of widespread models.
4. This final conclusion is of paramount importance: if oxygen vacancies display such a dominant role and they are present in every oxide, even though to different extents and with different concentrations and structures, the achievability of selectivity by chemoresisistive oxide sensors is deeply questionable.

On the other hand, the behavior of WO_3_ still appears to be far from elucidated, with the following possible issues to be solved and research directions to be pursued.

5. If direct extraction of oxygen from the WO_3_ surface by CO (Section 4) was a surprising result, even direct vacancy healing by NO_2_ remarkably differs from the widespread mechanism based on NO_2_ adsorption/desorption. It seems that such a mechanism still has to be reinforced by suitable measurements of the sensor exhaust during the NO_2_ tests.
6. In turn, such mechanistic conclusions indicate the need for a deep revision of the currently accepted models. This can only be done by intensifying the operando investigation of the sensor operation and the effective identification of the evolved species.
7. DFT modeling has been demonstrated as a powerful tool for verifying hypotheses about the surfaces and structures of WO_3_. The most commonly considered surface is the (001) plane in the γ-monoclinic phase, which is the most favorable and the most immediately formed upon cleaving single crystals. However, in a polycrystalline nanopowder, several other exposed facets are commonly present. In this sense, there are not many studies of the energy ordering [131] of the exposed crystal planes and of the oxygen vacancy formation energy. This topic needs to be investigated in detail to complete our knowledge of the surface properties of WO_3_. Other crystallographic phases, such as the orthorhombic phase, should also be considered.
8. Moreover, still there are very few DFT studies directly related to WO_3_ sensing. It would be helpful to obtain a catalogue of the most favorable adsorption sites for several gaseous analytes of interest. This is more and more desirable given the remarkably improved computing capabilities currently available.
9. If oxygen vacancies are vitally important in establishing the sensing behavior of WO_3_, then any improvement of WO_3_ sensors must be based on understanding and controlling such vacancies. “Understanding” means that a suitable catalogue of characterization techniques must emerge as a standard toolbox for establishing the vacancy concentration, topology and electronic structure. Therefore, XPS should ideally be complemented by other techniques, such as electron paramagnetic resonance, cathodoluminescence, etc. [152].
10. “Controlling” the vacancies means that the synthesis should be tailored toward the achievement of a given distribution of oxygen vacancies. Currently, treatments in reduced atmospheres are employed for preparing oxygen-deficient WO_3_. However, such structures are naturally amenable to being reoxidized during the sensor operation. Another strategy could be the designed introduction of dopants into the structure of WO_3_. For instance, let A_2_O_3_ be the oxide of a trivalent cation. The incorporation equation for substitutional defects for such an oxide into WO_3_ is, in Kröger–Vink notation:

$$A_{2}O_{3}\overset{{\mathrm{WO}}_{3}}{\to }2\;A_{W}^{\prime \prime \prime }+3\;V_{O}^{••}+3\;O_{O}$$

It can be seen that the concentration of the A cation may precisely pin the oxygen vacancies concentration. Therefore, the synthesis effort can be directed towards the achievement of a stable solid solution, given the suitable metal cations that can be incorporated into the WO_3_ structure. This is just a simple suggestion, mainly aimed to further show that cooperation between sensor science and other fields (in this case, structural chemistry) is more and more essential for understanding and enhancing the operation of WO_3_-based devices.

## Figures and Tables

**Table 1:** Summary of the main features of the WO_3_-based NO_2_ sensors described in Section 2.1.

Sample Morphology	Phase Composition	Gas Concentration	Response	Operating Temperature (°C)	Reference
Sintered powder	n/d	80 ppm	97	300	[32]
Lamellae	Monoclinic	500 ppb	>150	200	[34]
Nanoplates	Monoclinic	5 ppm	960	200	[35]
2D nanosheets	Monoclinic	50 ppb	~6	140	[36]
Porous nanosheets	Monoclinic	10 ppm	>450	100	[37]
Yolk–shell spheres	Monoclinic	50 ppb	~100	100	[38]
Nanosheets	Monoclinic	100 ppb	~50	75	[39]
Nanoplatelets	Monoclinic	1 ppm	~80	200	[40]
Nanotubes	Monoclinic	5 ppm	7	300	[41]
Nanoflowers	Monoclinic	50 ppb	>30	100	[43]
Nanocolumns	Monoclinic	10 ppm	22	110	[42]
Nanosheets	Monoclinic	40 ppb	30	150	[44]
Nanobricks	Monoclinic	100 ppm	12	300	[45]
Nanosheets	Monoclinic	4 ppm	>32	300	[46]
Nanoflowers	Monoclinic	100 ppm	>50	100	[47]
Nanosheets	Triclinic	300 ppb	18.8	100	[48]
Nanofibers	Monoclinic	50 ppm	>10^4^	150	[49]

**Table 2:** Summary of the main features of the WO_3_-based acetone sensors described in Section 2.2.

Sample Morphology	Phase Composition	Gas Concentration (ppm)	Response	Operating Temperature (°C)	Reference
Hollow spheres	Monoclinic	50	3.5	400	[53]
Thick films	Triclinic	50	4.56	300	[54]
Nanoplates	Triclinic	2	5	300	[55]
Nanoparticles	Monoclinic	10	10	350	[56]
Nanoplates	Monoclinic	300	50	307	[57]
Nanoplates	Monoclinic	500	40	200	[58]
Flower-like	Monoclinic	100	7	300	[59]
Nanotubes	Monoclinic	100	42.5	250	[60]
Urchin-like	Monoclinic	25	15	300	[61]
Nanoplates	Monoclinic	100	8	300	[62]
Nanosheets	Monoclinic	100	50	340	[63]
Nanosheets	Triclinic	1	2.04	230	[64]
Mesoporous nanofibers	Monoclinic	50	22	300	[65]
Nanosheets	Monoclinic	50	15	300	[66]
Urchin-like	Monoclinic	100	30	200	[67]

**Table 3:** Summary of the main features of the WO_3_-based ammonia and amines sensors described in Section 2.3.

Sample Morphology	Phase Composition	Gas Concentration (ppm)	Response	Operating Temperature (°C)	Reference
AMMONIA					
Sintered powders	Probably monoclinic	50	<5	200–600	[68]
Nanopowders	Monoclinic + triclinic	500	<5	350	[69]
Nanopowders	Monoclinic + triclinic	50	<6	250	[71]
Nanowires	Monoclinic	1500	9.7	250	[72]
Nanofibers	Orthorhombic	100	<5	300	[73]
Nanosheets	Hexagonal	100	35	350	[75]
Nanoplates	Monoclinic	100	<20	300	[76]
ALKYLAMINES (TEA/TMA)					
Hollow spheres	Orthorhombic	5	56.9	450	[77]
Nanosheets assembled in microspheres	Monoclinic	50	16	220	[78]
Hierarchical spheres	Monoclinic	10	35.3	150	[79]
Nanorods	Monoclinic	50	50	250	[80]

## Data Availability

Not applicable.
